# Supplementary material for: Effect of Exposure Concentration and Growth Conditions on the Association of Cerium Oxide Nanoparticles with Green Algae
Source: Nanomaterials (Basel). 2023 Sep 1;13(17):2468. doi: 10.3390/nano13172468 (PMC10490049; doi:10.3390/nano13172468)
Supplement: Supplementary file 1 [file nanomaterials-13-02468-s001.zip › nanomaterials-2508460-SI.pdf]

Effect of Exposure Concentration and Growth Conditions on the Association of Cerium Oxide Nanoparticles with Green Algae

Aiga Mackevica [1], Lyndsey Hendriks [2], Olga Meili-Borovinskaya [2], Anders Baun [1] and Lars Michael Skjolding [1,*]

[1] Department of Environmental and Resource Technology, Technical University of Denmark, Building 115, DK-2800 Kgs. Lyngby, Denmark
[2] TOFWERK, Schorenstrasse 39, 3645 Thun, Switzerland
* Correspondence: lams@dtu.dk

Supplementary Information

Table S1: Operating conditions for sc-ICP-QMS and sp-ICP-TOFMS

	Sc-ICP-QMS	Sc-ICP-TOFMS
Instrument	Nexion 350D (Perkin Elmer)	icpTOF S2 (TOFWERK)
Software	Syngistix v.2.5, Single Cell application	TOFpilot v.2.10 software, Particle module
Sample introduction system	Asperon™ spray chamber combined with syringe pump sample introduction	Single-Cell Sample Introduction System for ICP-MS (SC-SIS from Glass Expansion) combined with syringe pump (KD Scientific)
Sample flow [µL/min]	15	10
Nebulizer gas flow [L/min]	0.44	0.45
Make up gas flow [L/min]	0.7	0.8
Collision/reaction cell gas [mL/min]	3 (NH3) – only for ^{56}Fe measurements	5 (H_2/He mix)
Dwell times [ms]	0.1	1

Transport efficiency[1]	41%	65%

In order to ensure sufficient collection of cell events for reliable statistics, the samples were measured for 60s with 3 repeats for the QMS and for the TOFMS the samples were measured for 5 min with 2 repeats. 1% HNO_3 blanks and Milli-Q blanks were measured between the samples to check for carryover.

Cell tracer selection

In order to determine, which elements to use as cell "markers", the number of events for the different analytes (Mg, P, K, Mn and Fe) were recorded in the different samples and normalized with respect to the number of phosphorous events (see Figure S1, Figure S2 and Figure S3) and to Mn (Figure S4) and reproduced on independent days (see Figure S5).

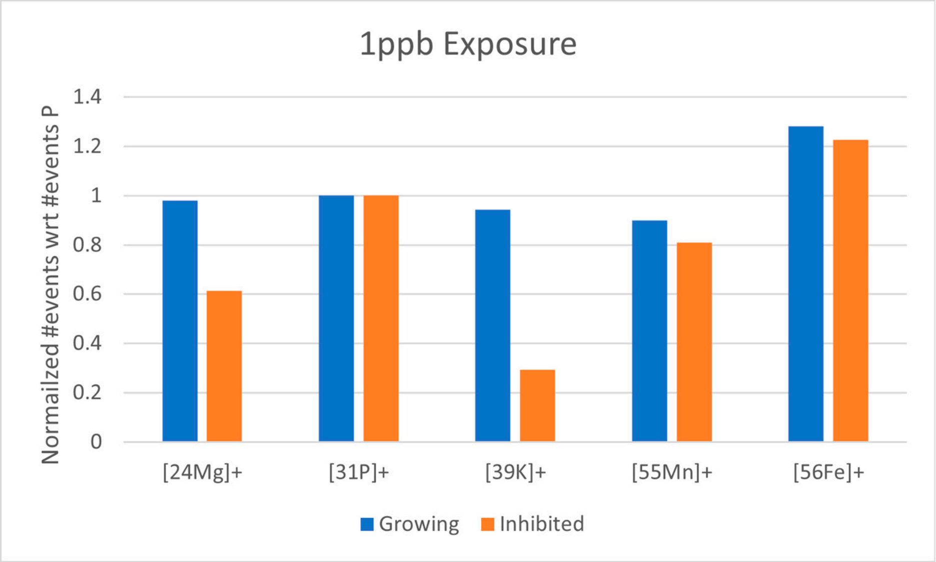

Figure S1: Normalization of the number of events recorded for the different analytes with respect to the number of P events, for both the growing control cells and the inhibited control cells at 1 μg/L CeO_2 NPs exposure.

[1] Transport efficiency was determined on the method deacribed by Pace et all. 2011 (Pace, H.E., Rogers, N.J., Jarolimek, C., Coleman, V.A., Higgins, C.P. and Ranville, J.F., 2011. Determining transport efficiency for the purpose of counting and sizing nanoparticles via single particle inductively coupled plasma mass spectrometry. *Analytical chemistry*, *83*(24), pp.9361-9369.)

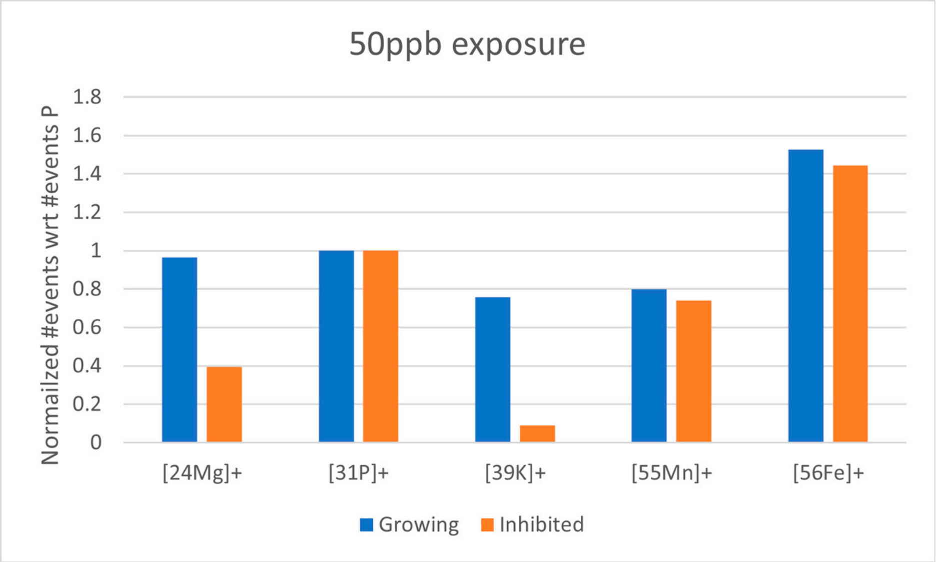

Figure S2: Normalization of the number of events recorded for the different analytes with respect to the number of P events, for both the growing control cells and the inhibited control cells at 50 μg/L CeO_2 NPs exposure.

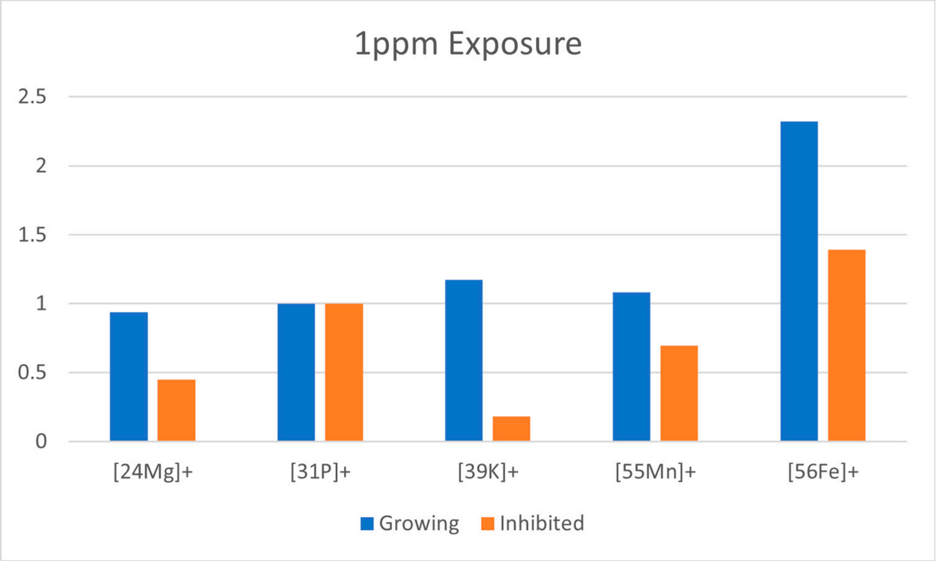

Figure S3: Normalization of the number of events recorded for the different analytes with respect to the number of P events, for both the growing control cells and the inhibited control cells at 1 μg/L CeO_2 NPs exposure.

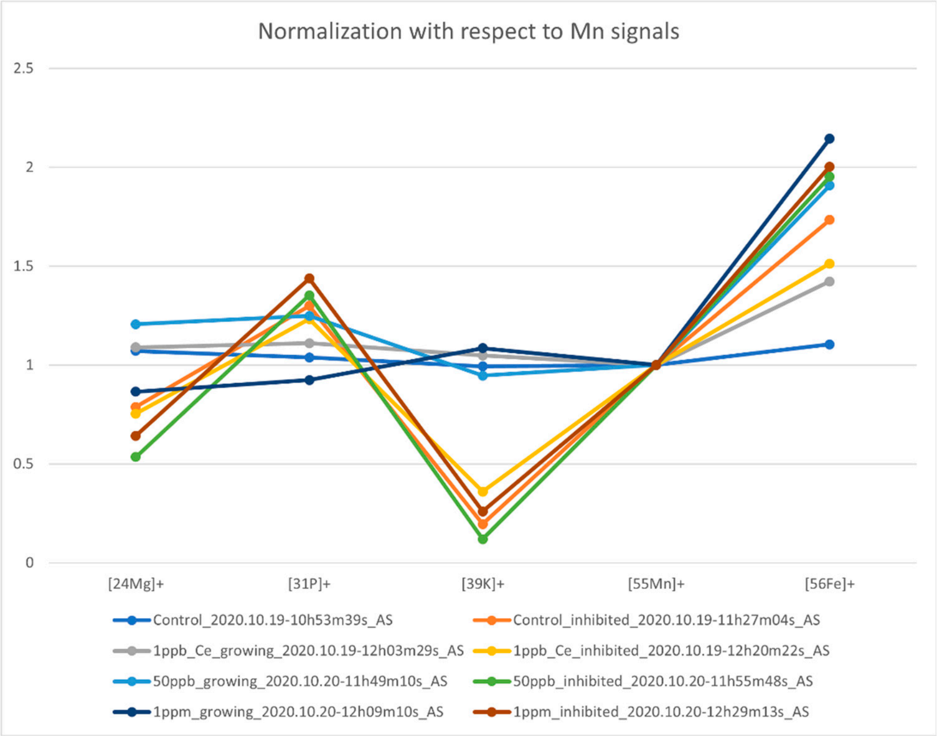

Figure S4: Normalization of the number of events recorded for the different analytes with respect to the number of Mn for the different samples. Overall, an increase in Fe signals can be observed with increasing exposure dose, which can be explained by oxidative stress.

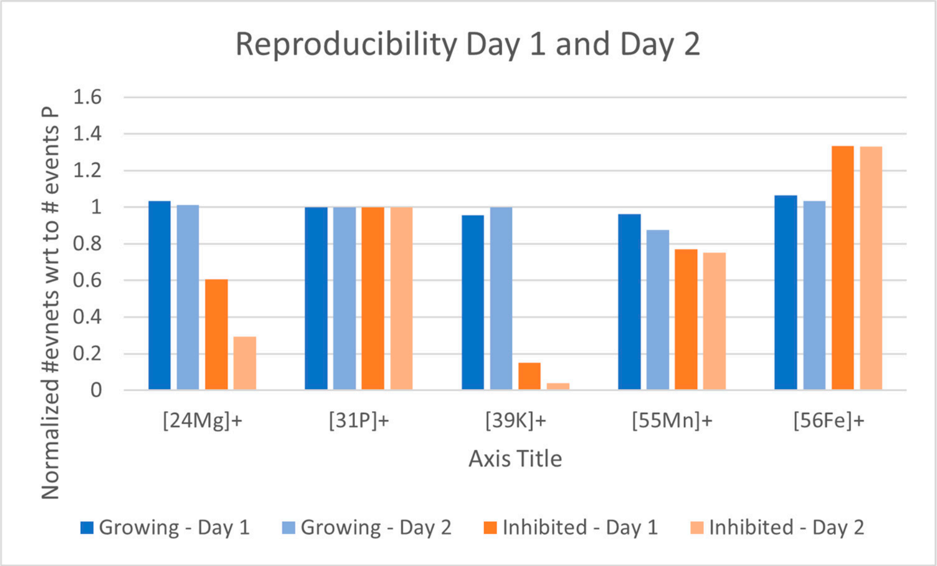

Figure S5: Display of the number of events recorded for the different analytes for both the growing control cells and the inhibited control cells on two subsequent days. The observed pattern between inhibited and growing cells is reproducible.

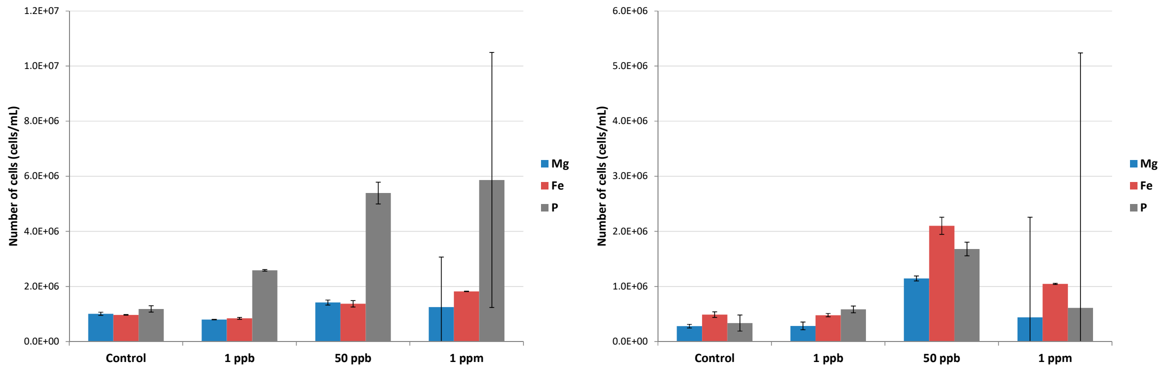

Figure S6 Cell counts per mL for growing (left) and inhibited (right) cells based on Mg, Fe and P tracing with sc-ICP-QMS.

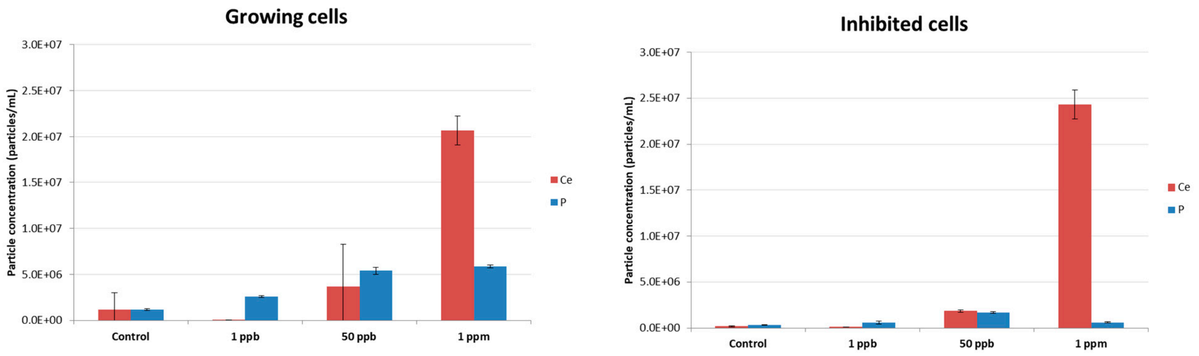

Figure S7: Number of events per mL of sample for Ce and P in growing and inhibited cells for different CeO_2 exposures.

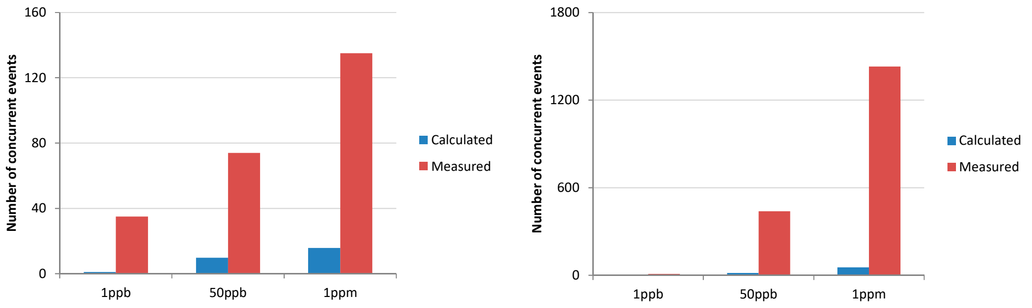

Figure S8: Predicted and measured number of concurrent events for growing (left) and inhibited (right) cells. Calculated number of concurrent events is based on Mn and Ce signals, Measured number of events is based on Ce, Mn and P simultaneous signal.

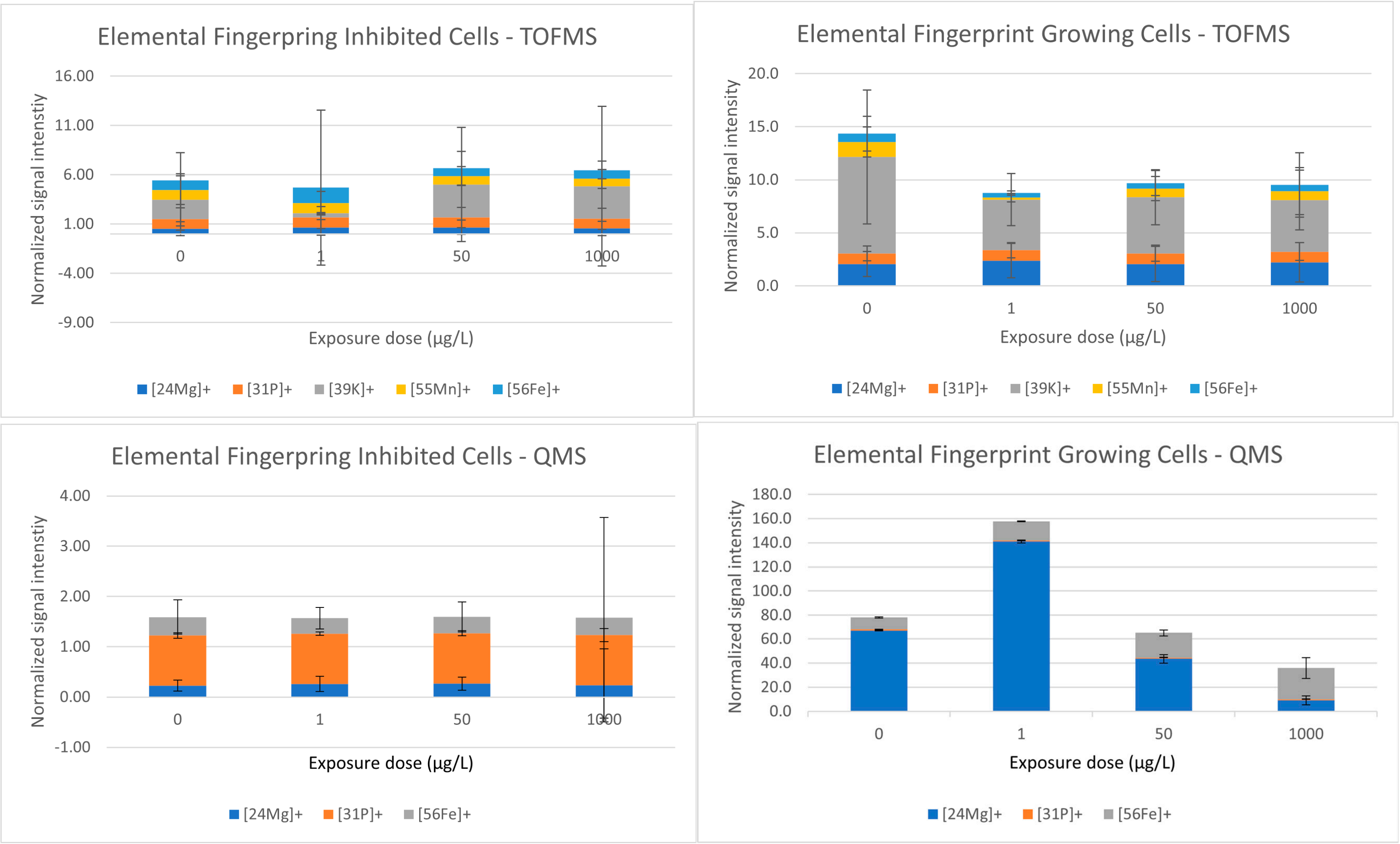

Figure S9: Normalized elemental cellular fingerprints for the freshwater green algae R. subcapitata measured by ICP-TOFMS (top row) and ICP-QMS (bottom row) for growing and inhibited populations. The error bars represent one standard deviation.
